# Effect of Solar Pre-Drying and Yeast Starter Inoculation Treatments on the Chemical Composition of Cocoa (*Theobroma cacao* L.) Beans from Southwestern Colombia

**DOI:** 10.3390/foods12244455

**Published:** 2023-12-12

**Authors:** Juan Florencio Tejeda, Jéssica Arango-Angarita, Jairo Leonardo Cuervo

**Affiliations:** 1 Food Science and Technology, Escuela de Ingenierías Agrarias, Universidad de Extremadura, Avda. Adolfo Suárez s/n, 06007 Badajoz, Spain; 2 Agronomy Department, National University of Colombia, Bogotá 111321, Colombia; yarangoa@unal.edu.co (J.A.-A.); jlcuervoa@unal.edu.co (J.L.C.)

**Keywords:** cocoa beans, pre-drying, yeasts, chemical composition

## Abstract

In Southwestern Colombia, cocoa clones are cultivated in which productivity characteristics predominate over bean quality. In this study, cocoa beans of the CCN-51 clone harvested in the Tumaco region (Nariño, Colombia) were fermented following four different treatments: (1) spontaneously (at room temperature for 120 h) in traditional conditions (Control); (2) traditional processing with a pre-drying (sun-dried for 24 h) treatment (PD); (3) with fermentation (for 120 h) after inoculation of a yeast starter culture (YS); and (4) including both treatments, pre-drying and yeast inoculation (PD + YS). Chemical composition, fatty acids, total polyphenol, methylxanthines (theobromine and caffeine) and lactic acid content of cocoa beans were determined. Chemical and fatty acid composition and theobromine content were not affected by the different fermentation treatments applied (*p* > 0.05). When analyzing total polyphenol content, YS (16.62 mg/g) and PD + YS (17.74) treatments significantly decreased (*p* < 0.05) the content of these compounds at the end of the fermentation process, affecting cocoa flavour, while PD treatment decreased (*p* < 0.05) the caffeine content (0.68 mg/g) of cocoa beans. Finally, lactic acid content decreased because of both inoculation of yeast starter (1.11 mg/g) and mainly the pre-drying treatment (0.60). In conclusion, solar pre-drying in the open air and the inoculation of yeast starter treatments could improve the final quality of cocoa beans.

## 1. Introduction

The highest production of cocoa (*Theobroma cacao* L.) in the world is located in the equatorial region of West Africa (about 75%), followed by the equatorial regions of Central and South America (about 20%) and the tropical parts of Asia (about 5%) [1]. According to the National Federation of Cacao, cocoa bean production in Colombia was 70,205 tons in 2021 [2]. In the region of Tumaco (Nariño, Colombia), in Southwestern Colombia, cocoa clones are cultivated in which productivity characteristics predominate over bean quality [3]. Such is the case of CCN-51 (Castro Naranjal Collection). This is a cocoa clone of Ecuadorian origin that is more resistant to common diseases, and it can produce yields greater than 4000 kg of dry beans/ha under high-density planting conditions and complete exposure to solar radiation [4].

Therefore, it is a crop of great importance worldwide. The pods obtained from these trees contain the seeds (commonly known as cocoa beans) from which chocolate is manufactured after a process of microbial fermentation and drying, which are the two main steps in the processing of cocoa beans [5]. Traditionally, cocoa bean fermentation is a spontaneous process. The mucilaginous pulp enclosing cocoa beans, which accounts for approximately 40% of the bean’s fresh weight, has a high content of fermentable sugars (glucose, fructose, sucrose) and a very acidic pH (3.0–3.5, mainly because of the presence of citric acid) [6]. This makes it an ideal medium for the growth of microorganisms naturally occurring at fermentation sites, including lactic and acetic acid bacteria and yeasts [7]. It is well known that both the quality of the fermented beans and the quality of the chocolate made from them are affected by the nature and distribution of these microorganisms present in the cocoa pulp [8]. Alcoholic fermentation of the pulp sugars by yeasts leads to the production of ethanol and its subsequent conversion into acetic acid via an exothermal reaction, which gradually increases the temperature of the fermenting seed mass, which can reach values close to 50 °C [9]. This causes endogenous changes in the beans that are fundamental in the development of a wide variety of other compounds with desirable and undesirable aromas, which are decisive in the quality of cocoa [10]. Furthermore, yeasts act by degrading cocoa proteins, with albumin and globulin being the predominant fractions, via the action of pectolytic enzymes, generating flavour precursors, namely free amino acids and peptides [10]. The fermentation process also contributes to the reduction in pulp and grain acidity via the utilization of citric acid [9].

The drying process of cocoa beans carried out after fermentation, aims to reduce the moisture content (from 55 to 60 to 8%) and the volatile acidity content to stop undesirable reactions and oxidation of polyphenolic compounds [11]. In this sense, sun drying is one of the most widespread drying methods in the rural areas of Colombia [12]. It is considered to be an adequate method for obtaining maximum flavour development [11]. Rodríguez-Campos et al. [13] reported that alcohols, esters, and pyrazine contents increased during the sun drying process. In contrast, acids, aldehyde, and ketone contents decreased. However, this method can result in the production of cocoa of heterogeneous quality due to climatic variations during drying and the increased labour required for processing [6]. So, the processing conditions of the cocoa beans, especially during fermentation and drying processes, are determining factors in the formation of the precursors and compounds responsible for the aroma of cocoa [14,15].

The objective of this study was to evaluate the effect of the solar pre-drying in the open air and the inoculation of yeast starter treatments on the physico-chemical composition of cacao (*Theobroma cacao* L.) beans (CCN51) cultivated in the Nariño-Tumaco region of Colombia.

## 2. Materials and Methods

### 2.1. Sampling

For this study, cocoa fruits of the clone CCN-51 were harvested using traditional methods in Tumaco (Nariño, Colombia) (01°29′03.6″ N latitude and 78°39′15.4″ W longitude), a geographical area with an altitude of 2 m a.s.l. Precipitation varies between 2265 and 6238 mm, with 168 to 264 days of rain per year, and the average annual temperature and relative humidity are 24 °C and 84.5%, respectively. Random sampling in farms from the above-mentioned area was carried out, and then the beans were extracted from the pod.

### 2.2. Cocoa Processing

Only healthy beans (seeds and placenta-free pulp) without any infection or physical damage were fermented in 40 × 40 × 50 cm wooden boxes divided into two compartments (each with 18.5 kg capacity) to facilitate the turning of the beans. The first turning was conducted after 48 h (anaerobic phase), and then it was repeated every 24 h to promote aerobic conditions until the end of the fermentation. The drawers presented bottom perforations to facilitate drainage of the exudations of the mucilaginous part of the fresh cocoa beans. Four different fermentation treatments were assayed: (1) Control—the beans were traditionally fermented at room temperature and spontaneously for 120 h; (2) Pre-drying treatment (PD)—the beans were previously sun-dried for 24 h under a greenhouse-type infrastructure. The beans were arranged in a layer thickness of 2 cm over a perforated surface 1 m above the floor. Then, the beans were traditionally fermented for 96 h; (3) Yeast starter treatment (YS)—the beans were inoculated with a mixed starter culture and fermented for 120 h; and (4) PD + YS treatment—the beans were previously sun-dried for 24 h and then treated with yeast starter culture and fermented 96 h more. In the four treatments, the beans were fermented in a closed room at an average ambient temperature of 27 °C. Finally, the beans were dried naturally in the sun in a wooden dryer, where the cocoa was arranged in a thin layer for 5 days (until reaching a final moisture content under 7%). The dried beans were vacuum packed and shipped to Spain, where they were maintained at –80 °C until analyses. In this study, four replicates per treatment were performed, and the mean values were reported.

### 2.3. Preparation of the Starter Culture

The yeast starter culture was composed of *Saccharomyces cerevisiae*, *Wickerhamomyces anomalus*, *Candida tropicalis*, and *Pichia kudriavzevii*, previously isolated from spontaneous cocoa fermentations in the Tumaco region. Yeasts were propagated in YPD broth (10 g yeast extract, 20 g bactopeptone, and 20 g dextrose, per litre distilled water). Following propagation, cell densities were determined according to the McFarland method. Each experimental unit (wooden box) was inoculated with 15 mL of the liquid starter culture (cell density was 1.2 × 10^9^ cell/mL).

### 2.4. Chemical and Fatty Acid Composition Analysis

Moisture (oven air-drying method), total protein (Kjeldahl nitrogen), total fat (Soxhlet method), total dietary fibre (enzymatic–gravimetric determination), and ash (muffle furnace) were analyzed following official methods [16]. Carbohydrates were calculated by difference. Fatty acid methyl esters from the lipids obtained were prepared by acidic-trans-esterification in the presence of sodium metal (0.1 N) and sulphuric acid (5% sulphuric acid in methanol) [17], and they were analyzed via gas chromatography, using a Hewlett-Packard HP-4890 Series II gas chromatograph (Hewlett-Packard, Palo Alto, CA, USA) equipped with a split/splitless injector and a flame ionization detector (FID). Fatty acids were separated on a nitroterephthalic acid modified polyethylene glycol (HP-FFAP) semi-capillary fused silica column (30 m long, 0.53 mm i.d., and 1 μm film thickness; Hewlett-Packard, Palo Alto, CA, USA) maintained at 230 °C for 25 min. Injector and detector temperatures were 250 °C. The carrier gas was nitrogen at 1.8 mL/min. Individual fatty acids were identified by comparison of their retention times with those of standard reference mixtures (Sigma Chemical Co., St. Louis, MO, USA). Results were expressed as the percentage of total fatty acids present. 

### 2.5. Determination of Total Polyphenol Content

The total polyphenol content (TPC) of cocoa extracts was determined spectrophotometrically using the Folin–Ciocalteu reagent according to the method of Cros et al. [18]. Briefly, 0.5 g of ground cacao was mixed with 40 mL of acetone/water mixture (80:20) and filtered through Whatman No. 1 filter paper (Whatman, Brentford, UK). The residue was washed with 20 mL of the same acetone/water mixture, and the total volume of filtrate was made up to 100 mL in a volumetric flask. One mL of the previously prepared extract, 7 mL of distilled water, and 500 μL Folin–Ciocalteu’s reagent (Sigma-Aldrich^®^, St. Louis, MO, USA) were mixed. After 3 min, 1 mL of saturated Na_2_CO_3_ solution was added. Then, the mixture was incubated for 2 h, and the absorbance was measured at 760 nm against a blank sample using a digital spectrophotometer (Thermo Fisher Scientific Inc., Waltham, MA, USA). A calibration curve with gallic acid (Sigma-Aldrich^®^, St. Louis, MO, USA) as the standard with six known concentrations was prepared. The Least Squares method was used to calculate the linear regression and correlate absorbance measures in cocoa samples with polyphenol content. The TPC was expressed as a milligram equivalent of gallic acid (GAE) per gram of dried cocoa sample (mg GAE/g dry cocoa). All measurements were performed in duplicate.

### 2.6. Analysis of Methylxanthines and Lactic Acid

The extract for theobromine, caffeine, and lactic acid determination by HPLC was obtained according to the method described by Ramos et al. [10], with modifications. One gram of dried and ground cocoa sample was mixed with 20 mL distilled water by vigorous vortexing for 5 min. The mixture was centrifugated at 7000 rpm at 4 °C for 10 min. The supernatant was collected, and the precipitate was resolved with 5 mL distilled water, vortexed, and centrifugated as previously described. The two supernatant fractions were mixed and made up to 25 mL, and then 2 mL were taken and filtered through a 0.22 µm Millipore filter (Millipore, Bedford, UK) to carry out the HPLC analysis.

HPLC analyses were carried out via high-performance liquid chromatography using an Agilent 1100 Series (Agilent Technologies, Palo Alto, CA, USA) system with a diode array detector (Agilent 1200 series) set at 280 nm. Separation was performed on a Lichrospher 100 RP-18 column (250 mm long, 4 mm id, and 5 µm particle size) with a mobile phase of methanol/acetic acid/water (79:1:20), an injection volume of 50 µL, and a flow rate of 1.0 mL/min. Theobromine, caffeine, and lactic acid were identified by comparing the retention times to those of standards. Quantitative analyses were carried out using the individual standard curves for each compound determined.

### 2.7. Statistical Analysis

Data from chemical, fatty acid, total polyphenol, methylxanthines, and lactic acid compositions are presented as the mean and standard deviation (SD). A one-way analysis of variance (ANOVA) was performed to check the differences between cocoa bean treatments (Control, PD, YS, and PD + YS). The differences between the means of each treatment were significant for less than 5% of the total variance (*p* < 0.05) and were compared using the Tukey test. The statistical analyses were performed by using the Minitab 17 statistical software (version 17.2.1, Minitab Pty Ltd., Sydney, Australia).

## 3. Results and Discussion

### 3.1. Chemical Composition of Cocoa Bean Samples

The chemical composition and the effect of processing treatment of unroasted CCN-51 cocoa bean samples after fermentation and drying processes are presented in Table 1. Moisture content ranged from 4.9 to 5.2 g/100 g, while the contents of the two main components of the samples, fat and carbohydrate components, varied from 39.0 to 41.4 and 38.2 to 39.2 g/100 g, respectively. Results in the literature confirm that fat is the main component of cocoa beans, followed by carbohydrates [19]. With respect to protein content, the results obtained varied between 12.6 and 14.0 g/100 g. Finally, ash content was between 2.7 and 2.9 g/100 g. These results agree with previous data obtained in other studies of cocoa beans [19,20]. In the present experiment, neither the PD, YS, nor the PD + YS treatment had any significant effect (*p* > 0.05) on the chemical composition of cocoa beans compared to the Control treatment. It is well documented that both fermentation and subsequent drying of the beans (artificially or by sun-drying) affect their physico-chemical composition [14]. In this sense, Barrientos et al. [15] observed a decrease in the total fat content of cocoa beans fermented and sun-dried for 132 h. In the same way, protein content decreases during pod storage and cocoa bean fermentation. This is caused by the endogenous breakdown of cocoa bean proteins into oligopeptides and free amino acids [21]. This reduction is reported to be due to the action of protease enzymes in the pods during storage, and these enzymes initiate the process of proteolysis [22]. De Brito et al. [23] also pointed out a decrease in protein content during the cocoa bean fermentation process, but it remained constant during the subsequent sun-drying process. In our study, there was no observed effect from the pre-drying treatment on the physico-chemical composition of the beans. This was probably due to the shorter pre-drying process (24 h) carried out in our experiments compared to other studies [23]. In the same way, the chemical composition of cocoa beans was not affected by yeast inoculation in the present experiments. Other authors revealed differences in both visual and other sensory characteristics, as well as chemical composition, between beans fermented in the absence and presence of yeasts [24]. In our study, no effect of yeast treatment was observed on the carbohydrate content of cocoa beans. Several studies showed that pulp sugars, mainly non-reducing sugars, are the main substrates for microbial fermentation during the fermentation process of cocoa beans. However, the same studies found that during the fermentation process, as the number of days of processing increases, there is an increase in reducing sugars due to the inversion of sucrose [23,25], which could explain the absence of differences in total carbohydrate content at the end of cocoa processing in our study. This creates environmental conditions that produce flavour precursors within the beans [7]. So, yeast growth or inoculation is essential to develop cocoa bean fermentation [24].

### 3.2. Fatty Acid Composition of Pre-Dried and Yeast Starter Inoculated Cocoa Bean Samples

The results related to the fatty acid composition of cocoa beans are shown in Table 2. The main fatty acids were C18:0 (33.6–33.8%), C18:1 (32.2–32.4%), and C16:0 (29.2–29.3%). These results are in accordance with those obtained by other authors [19,20], who also reported that these three fatty acids account for more than 90% of the total fatty acids identified in cocoa butter. Total saturated fatty acids (SFA) showed the highest content (up to 64%), followed by total monounsaturated fatty acids (MUFA) (up to 32%), and finally, total polyunsaturated fatty acids (PUFA) (less than 3%). The ratio of saturated/unsaturated (S/U) varied from 1.82 to 1.85. In this study, no significant differences (*p* > 0.05) in any of the fatty acids identified (nor in total SFA, MUFA, PUFA, and S/U ratio) were found between the Control and the different treatments conducted. Fatty acid profiles in cocoa beans are mainly affected by the variety and the growing conditions of the cocoa beans [20]. However, other studies confirm very small differences in the fatty acid composition of cocoa from different geographical origins [19,20]. In accordance with our results, no studies found changes in the fatty acid profile of cocoa beans because of different fermentation or pre-drying processes. So, our results confirm that during pre-drying or fermentation of cocoa, by adding a yeast culture, no biochemical reactions are generated that could modify the fatty acid profile of the dried cocoa beans. Differences in fatty acid composition, mainly MUFA and PUFA, between treatments can influence the final cocoa butter quality. In this sense, the presence of oleic acid in high quantities plays an important role because it influences the type of triacylglycerol formed, affecting the melting point of cocoa butter and therefore the final quality of the manufactured product [19].

### 3.3. Total Polyphenol, Methylxanthine, and Lactic Acid Content of Pre-Dried and Yeast Starter Inoculated Cocoa Bean Samples

Cocoa fermentation conditions, as well as other post-harvest processing operations such as roasting of cocoa beans, affect both the concentration of polyphenols and methylxanthines, thus affecting the quality of the final product [26]. The kernel or cotyledon of cocoa beans is rich in polyphenols and alkaloids, including caffeine and theobromine, stored in a single vacuole from polyphenolic cells [27]. Polyphenols and alkaloids are mainly responsible for the flavour of the bean (polyphenols contribute to the flavour and colour of cocoa beans, and alkaloids to the bitterness) [28], and therefore they confer the characteristic flavours and aromas of chocolate [27]. The results of total polyphenol, theobromine, caffeine, and lactic acid contents of unroasted cocoa bean samples from the different treatments studied are shown in Table 3. The total polyphenol content ranged from 16.6 to 19.3 mg gallic acid/g. These contents are in accordance with those reported by Batista et al. [29] but slightly lower than those reported in beans by other authors (34–60 mg/g) [30]. These differences could be related to the effect that both genotypic characteristics and the geographical area of cocoa production have on the total polyphenol concentration of the cocoa bean [31]. In the present study, significant differences (*p* < 0.05) between treatments were detected. Control and PD treatments showed higher contents (18.37 and 19.25 m/g, respectively) than the YS treatment (16.62 m/g), and PD + YS showed intermediate contents (17.74 m/g). These results show that the pre-drying treatment, prior to the start of the cocoa fermentation process and carried out under natural conditions for 24 h, did not affect the final polyphenol content of the cocoa bean. Temperature and drying duration are determining factors in the degradation of polyphenols. Polyphenol content decreases with increasing temperature above 60 °C, as reported by Hii et al. [5]. But also, at long drying times, irreversible oxidative processes of polyphenols can take place above 40 °C [32]. This may be caused by oxidase reactions, both non-enzymatic and catalyzed by polyphenol oxidase, during the drying process. However, the temperatures reached during pre-drying in our study and the short time during which drying takes place did not seem to be sufficient to contribute to a decrease in the total polyphenol content compared to the Control treatment. In contrast, inoculation of selected culture yeast on cocoa beans significantly decreased the total polyphenol content at the end of the fermentation process when compared to those cocoa beans fermented spontaneously. This fact could be related to the cell damage caused by organic acids, facilitating the contact between the enzyme and substrate [8]. In agreement with our results, several studies have reported an increase in the total phenolic content of cocoa spontaneously fermented compared to cocoa beans fermented after culture yeast inoculation [29]. This seems to be related to the effect that the starter culture may have on the microorganisms responsible for spontaneous fermentation. Although polyphenols are valuable antioxidants, their presence has been linked to astringency and bitterness in cocoa and cocoa products [28]. Consequently, the inoculation of cocoa beans with defined yeast starter cultures could affect the flavour quality of cocoa.

With respect to methylxanthines analyzed, no significant differences were observed in theobromine content between treatments, with an average value of 7.38 mg/g. This content agrees with that reported by other authors [29]. The theobromine content in cocoa beans seems to be unaffected by cocoa fermentation conditions, including spontaneous fermentation or inoculation with starter cultures [29], in accordance with our findings. However, the caffeine content exhibited significant differences due to treatment, showing PD lower (*p* < 0.05) content (0.68 mg/g) than Control, YS, and PD + YS treatments (1.02, 0.93 and 0.98 mg/g, respectively). The present results showed that the pre-drying treatment of cocoa beans in the sun for 24 h decreased the caffeine content. In contrast, inoculation of cocoa beans with a yeast culture did not affect the caffeine content. These results agree in part with other studies which indicated that caffeine content in cocoa beans is affected by genotype, processing conditions and spontaneous or inoculated fermentation [29]. Finally, the lactic acid amount varied between 0.60 and 1.40 mg/g, with Control showing higher content than YS, and this last one higher than PD and PD + YS treatments (*p* < 0.001). These results evidenced that both PD and YS treatments cause a decrease in the lactic acid concentration of cocoa beans as a consequence of a partial reduction in cocoa pulp, compared to traditional and spontaneous fermentation. In our study, the mucilage lost about 20% of the initial weight during the solar pre-drying period. This is in accordance with other results reported previously [9]. It has been well documented that excessive cocoa bean pulp leads to an increase in the sourness of the cocoa beans and thus a decrease in the flavour quality, mainly due to the high production of acid during fermentation [7]. Both treatment processes investigated in this study, solar pre-drying and yeast starter inoculation, are responsible for the reduction in part of the cocoa bean pulp in two ways, namely mechanically and enzymatically, respectively [22]. Kongor et al. [33] reported that mechanical depulping via spreading beans onto a flat surface and exposing them to the sun for several hours leads to an increase in the sweating produced during fermentation. This is due to bruising of the beans and their cellular structures, which activates the cellular enzymes. In the same way, the reduction in bean acidity during fermentation in cocoa beans inoculated with a defined mixed starter culture, compared to those from spontaneous fermentation, could be related to the enzymatic activity of the yeasts [34].

## 4. Conclusions

This study showed that the chemical and fatty acid composition of cocoa beans were not affected by a pre-drying treatment and a subsequent fermentation process with a starter culture of selected yeasts, compared to the traditional process by spontaneous fermentation, carried out by regional farmers in Tumaco-Nariño region in Colombia. Fermentation carried out after inoculation of a defined starter culture of yeasts caused a decrease in total polyphenol content, which could contribute to improving the flavour quality of cocoa. There was a noticeable decrease in lactic acid in cocoa beans. This was caused by fermentation treatment with selected yeasts and especially by a pre-drying treatment with the sun. This would reduce the final acidity of the cocoa and improve the final quality of the cocoa manufactured into chocolate, given that excessive acidification of the cocoa beans during fermentation is associated with a lower sensory quality of the cocoa. So, it can be concluded that solar pre-drying combined with yeast starter inoculation could be an interesting way to improve cocoa bean quality. 

## Figures and Tables

**Table 1:** Chemical composition (g/100 g) of unroasted CCN-51 cocoa bean samples from the different treatments.

	Control	PD	YS	PD + YS	*p*
Moisture	4.98 ± 0.45	5.08 ± 0.28	5.05 ± 0.29	5.21 ± 0.32	0.607
Total protein	12.65 ± 0.32	13.98 ± 0.49	13.84 ± 0.54	13.72 ± 0.64	0.564
Total fat	41.38 ± 1.17	39.06 ± 0.82	39.01 ± 1.01	39.28 ± 0.99	0.409
Carbohydrate	38.25 ± 0.34	39.07 ± 0.42	39.17 ± 0.39	39.00 ± 0.17	0.339
Fibre, total dietary	15.31 ± 0.28	16.28 ± 0.51	16.01 ± 0.48	14.94 ± 0.41	0.268
Ash	2.74 ± 0.21	2.81 ± 0.19	2.93 ± 0.24	2.79 ± 0.17	0.249

Values expressed as means ± standard deviation. Composition analysis of four samples. *p*: the means from the four treatments are significantly different for *p* < 0.05 according to Tukey’s test. Control: traditional treatment. PD: traditional treatment with pre-drying treatment. YS: treatment with inoculation of yeasts. PD + YS: traditional treatment with pre-drying treatment and inoculation of yeasts. Carbohydrates were calculated by difference.

**Table 2:** Fatty acid composition (% of total fatty acids) of unroasted CCN-51 cocoa bean samples from the different treatments.

	Control	PD	YS	PD + YS	*p*
C14:0	0.08 ± 0.01	0.08 ± 0.01	0.08 ± 0.02	0.08 ± 0.01	0.650
C15:0	0.05 ± 0.02	0.05 ± 0.01	0.04 ± 0.01	0.05 ± 0.02	0.697
C16:0	29.26 ± 0.44	29.18 ± 0.52	29.24 ± 0.41	29.20 ± 0.53	0.952
C16:1	0.25 ± 0.02	0.26 ± 0.01	0.27 ± 0.03	0.26 ± 0.02	0.304
C17:0	0.30 ± 0.02	0.29 ± 0.04	0.30 ± 0.01	0.32 ± 0.02	0.754
C17:1	0.02 ± 0.01	0.03 ± 0.01	0.03 ± 0.01	0.03 ± 0.01	0.755
C18:0	33.81 ± 0.91	33.63 ± 0.87	33.75 ± 1.01	33.84 ± 0.92	0.835
C18:1	32.22 ± 0.81	32.37 ± 0.77	32.26 ± 0.94	32.22 ± 0.83	0.546
C18:2	2.42 ± 0.11	2.50 ± 0.19	2.41 ± 0.09	2.41 ± 0.07	0.342
C18:3	0.19 ± 0.02	0.20 ± 0.03	0.19 ± 0.02	0.20 ± 0.02	0.846
C20:0	1.18 ± 0.04	1.18 ± 0.05	1.20 ± 0.04	1.17 ± 0.03	0.737
C20:1	0.04 ± 0.03	0.05 ± 0.02	0.04 ± 0.01	0.05 ± 0.03	0.686
C22:0	0.18 ± 0.03	0.18 ± 0.01	0.17 ± 0.05	0.17 ± 0.02	0.808
SFA	64.86 ± 0.86	64.59 ± 1.04	64.78 ± 0.80	64.83 ± 0.92	0.567
MUFA	32.53 ± 0.93	32.71 ± 0.89	32.6 ± 0.99	32.56 ± 0.94	0.563
PUFA	2.61 ± 0.11	2.70 ± 0.14	2.60 ± 0.18	2.61 ± 0.10	0.370
UFA	35.14 ± 0.99	35.41 ± 1.02	35.20 ± 0.98	35.17 ± 0.96	0.445
S/U	1.85 ± 0.12	1.82 ± 0.09	1.84 ± 0.08	1.84 ± 0.10	0.193

Values expressed as means ± standard deviation. *p*: the means from the four treatments are significantly different for *p* < 0.05 according to Tukey’s test. Control: traditional treatment. PD: traditional treatment with pre-drying treatment. YS: treatment with inoculation of yeasts. PD + YS: traditional treatment with pre-drying treatment and inoculation of yeasts. SFA: saturated fatty acids. MUFA: monounsaturated fatty acids. PUFA: polyunsaturated fatty acids. UFA: unsaturated fatty acids. S/U: saturated/unsaturated fatty acids ratio.

**Table 3:** Total polyphenol content (mg gallic acid/g dry cocoa) and theobromine, caffeine, and lactic acid contents (mg/g dry cocoa) of unroasted CCN-51 cocoa bean samples from the different treatments.

	Control	PD	YS	PD + YS	*p*
Total polyphenols	18.37 ± 0.47 ^a^	19.25 ± 0.52 ^a^	16.62 ± 0.39 ^b^	17.74 ± 0.28 ^ab^	0.015
Theobromine	7.29 ± 0.33	7.48 ± 0.25	7.45 ± 0.16	7.29 ± 0.23	0.195
Caffeine	1.02 ± 0.18 ^a^	0.68 ± 0.07 ^b^	0.93 ± 0.17 ^a^	0.98 ± 0.13 ^a^	0.049
Lactic acid	1.40 ± 0.04 ^a^	0.60 ± 0.17 ^c^	1.11 ± 0.21 ^b^	0.60 ± 0.18 ^c^	0.000

Values expressed as means ± standard deviation. *p*: the means from the four treatments are significantly different for *p* < 0.05 according to Tukey’s test. a, b, c Values within a row with different superscripts differ significantly. Control: traditional treatment. PD: traditional treatment with pre-drying treatment. YS: treatment with inoculation of yeasts. PD + YS: traditional treatment with pre-drying treatment and inoculation of yeasts.

## Data Availability

Data are contained within the article.
